# Actual long-term survival after resection of stage III soft tissue sarcoma

**DOI:** 10.1186/s12885-020-07730-3

**Published:** 2021-01-05

**Authors:** Do Weon Lee, Han-Soo Kim, Ilkyu Han

**Affiliations:** 1 Department of Orthopaedic Surgery, Seoul National University Hospital, Seoul, South Korea; 2 Department of Orthopaedic Surgery, Seoul National University College of Medicine, Seoul, South Korea

**Keywords:** Sarcoma, Soft tissue sarcoma, Survival

## Abstract

**Background:**

Actuarial survival based on the Kaplan–Meier method can overestimate actual long-term survival, especially among those with factors of poor prognosis. Patients with American Joint Committee on Cancer stage III soft tissue sarcoma (STS) represent a subset with a high risk of STS-specific mortality. Therefore, we aimed to characterize the clinicopathological characteristics associated with actual long-term survival in patients with stage III STS.

**Methods:**

We retrospectively reviewed 116 patients who underwent surgical resection for stage III STS with curative intent between March 2000 and December 2013. Long-term survivors (*n* = 61), defined as those who survived beyond 5 years, were compared with short-term survivors (*n* = 36), who died of STS within 5 years.

**Results:**

Multivariate logistic regression analyses showed that a tumor size < 10 cm [odds ratio (OR) 3.95, *p* = 0.047], histological grade of 2 (OR 8.12, *p* = 0.004), and American Society of Anesthesiologists (ASA) score of 1 (OR 11.25, *p* = 0.001) were independently associated with actual 5-year survival. However, 66% of the long-term survivors exhibited factors of poor prognosis: 36% had a tumor size > 10 cm and 48% had a histological grade of 3. Leiomyosarcoma (3 of 10) was negatively associated with actual long-term survival.

**Conclusions:**

Actual 5-year survival after resection of stage III STS was associated with tumor size, histological grade, and ASA score. However, majority of the actual 5-year survivors exhibit factors of poor prognosis, suggesting that aggressive treatment should be offered for a chance of long-term survival in these patients.

## Background

Although soft tissue sarcomas (STSs) are rare, representing less than 1% of all adult solid malignant cancers, [1, 2] they may confer high mortality due to delayed diagnosis and advanced disease at presentation [3]. Early-stage STS lacks distinctive symptoms, hindering early diagnosis. Additionally, compared to other cancers, STS tends to occur more frequently in young adults and adolescents, and their loss of years may be more devastating [4, 5]. Moreover, the incidence of STS has increased by more than 20% over the past 2 decades, although this may be due to improved surveillance [1, 6]. Therefore, it is important to characterize the factors related to the prognosis of STS and provide treatment accordingly.

Many studies have been conducted regarding the factors associated with the prognosis of STS. Tumor size, histological grade, and metastasis are well-established prognostic factors and comprise the most commonly used American Joint Committee on Cancer (AJCC) staging system. However, certain histological subtypes, microscopic positive surgical margins, and even some molecular parameters are also related to adverse disease-specific survival [7, 8]. A more recent study suggested that age, race, the duration of symptoms, the anatomical location, and administration of radiotherapy are also important prognostic factors of disease-specific mortality [9–11]. Surprisingly, despite recent advances in STS treatment and surveillance, there seems to be little improvement in the survival rate [12, 13]. The 5-year estimated survival rates for stages I, II, III, and IV STS are approximately 90, 70, 50, and 10% to 20%, respectively [14].

The outcome of most cancer survival analyses is actuarial survival based on the Kaplan–Meier method, which includes censored data and estimates long-term survival. Actuarial survival can overestimate actual long-term survival, especially among patients with poor prognosis [15]. Moreover, since most of the established prognostic factors of STS are derived from actuarial data, whether these risk factors truly preclude actual long-term survival has not been elucidated.

Patients with AJCC stage III STS represent a subset with a high risk of STS-specific mortality. To date, the clinicopathological characteristics of actual long-term survivors of AJCC stage III STS have not been well studied. Therefore, we aimed to characterize the clinicopathological characteristics associated with actual long-term survival in patients with AJCC stage III STS.

## Methods

### Patients

**Fig. 1:**
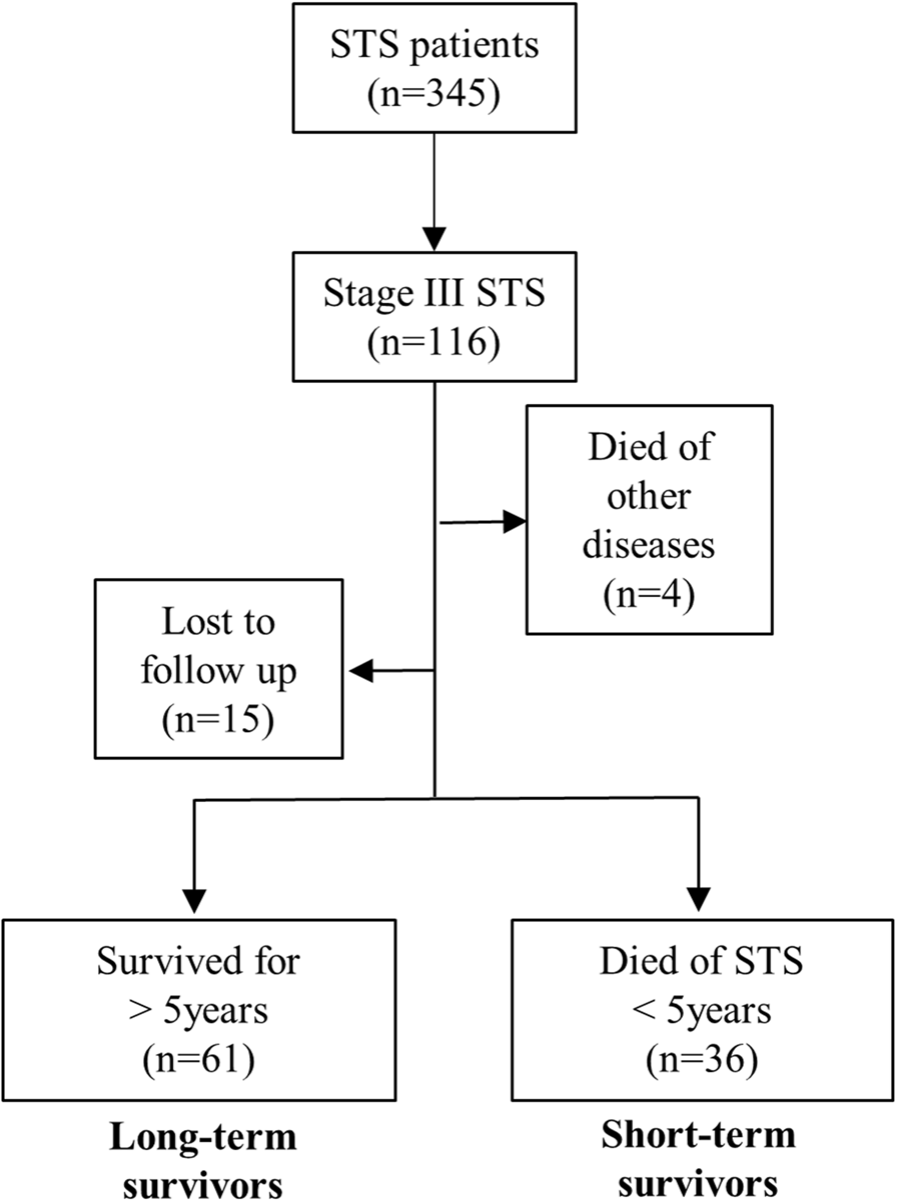
Flow diagram of patients screened and grouped; STS, soft tissue sarcoma

One hundred and sixteen patients who underwent resection for AJCC stage III STS at our institute between 2000 and 2013 were reviewed. Of the 116 patients, 4 died of other causes, and 15 were lost to follow-up within 5 years. After excluding these 19 patients, 97 patients were included in the final analyses (Fig. 1). The median follow-up duration in the entire cohort was 5.0 years (range, 0.3–17.3 years). Patients who survived beyond 5 years were considered long-term survivors. The Institutional Review Board of our institute approved this study and exempted of the informed consents (no. H-1991-117-1080).

### Clinicopathological variables

To compare the clinicopathological characteristics of long-term and short-term survivors, medical records were reviewed for patient, tumor, and treatment characteristics. Regarding the patient characteristics, we evaluated the patients’ age at the time of resection, sex and preoperative American Society of Anesthesiologists (ASA) score [16].

Regarding the tumor characteristics, we analyzed the tumor size, histological type, histological grade, and initial presentation status. The tumor size was subdivided into two groups: 5–10 cm and > 10 cm. The histological classification was based on the guidelines of the 2013 World Health Organization classification of soft tissue tumors [17]. The histological grade was evaluated according to the Fédération Nationale des Centres de Lutte Contre Le Cancer (FNCLCC) system [18]. Patients who presented after inappropriate excision were categorized into the unplanned excision group.

The treatment characteristics included the surgical margin, histological margin, chemotherapy, and radiotherapy. The surgical margins were classified as either marginal or wide according to the Enneking classification [19]. A wide margin was accomplished when the tumor was removed with a surrounding cuff of normal tissue. Regarding the histological margin, the presence of microscopic tumor cells at the resection margin was considered an R1 margin. For surveillance for distant metastasis, chest CT was performed every 3–4 months for 2 years, then every 6 months for the next 3 years, and then annually until post-operative 10 years. Imaging of the primary site was performed using magnetic resonance imaging (MRI) or ultrasonography (US) based on the risk of local recurrence.

### Statistics

Continuous variables are presented as medians and ranges, while categorical variables are presented as frequencies and percentages; these were compared using the Wilcoxon rank test and Fisher’s exact test, respectively. To analyze the clinicopathological factors associated with actual long-term survival, univariate and multivariate analysis was performed using logistic regression. Factors of significance (*p* < 0.05) in univariate analyses were included in the multivariate analysis. Additionally, Kaplan-Meier curves of the two groups were drawn to compare actuarial survival rate with actual survival. Statistical analyses were performed using SPSS version 25.0.0 (IBM Inc., Armonk, New York). A *p* value < 0.05 was considered significant.

## Results

### Actual long-term survival

**Fig. 2:**
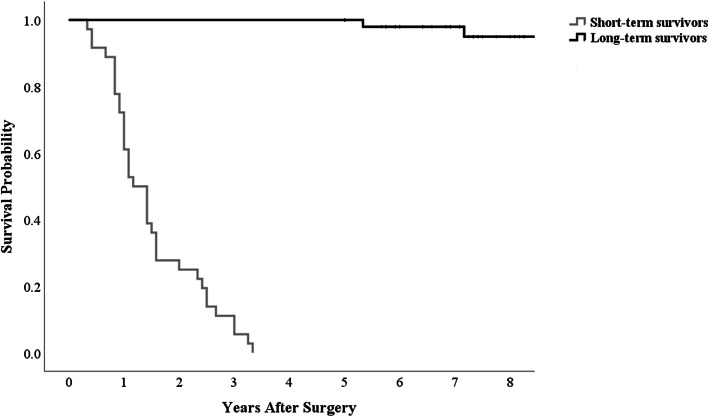
Estimated survival curves of long-term and short-term survivor groups in the study

Among the 97 patients, 61 patients (62.9%) survived for more than 5 years. The median follow-up duration of the 61 long-term survivors was 7.3 years (range, 5.0–17.3 years). Among the 61 patients, 5 patients developed local recurrence at 9, 14, 25, 33, and 127 months postoperatively, and 2 patients presented with metastases at 15 and 44 months postoperatively. One patient, whose recurrence was identified at 127 months, revisited the clinic with a symptomatic lump and subsequently diagnosed with local recurrence with MRI and biopsy. Of these 7 patients, one patient died at postoperative 12.5 years due to cancer progression. The median survival of the 36 short-term survivors was 1.3 years (range, 0.3–3.3 years). The Kaplan–Meier survival curves of the two groups (long-term and short-term survivors) are shown in Fig. 2 (*p* < 0.001). The actuarial 5-year overall survival rate using the Kaplan –Meier method was 62.8%. The actuarial relapse-free survival rate was slightly higher than the actual rate (60.9% vs. 56.7%).

### Comparison of characteristics between long-term survivors and short-term survivors

When the clinicopathological characteristics of the 61 patients who survived for more than 5 years and 36 patients who died within 5 years were compared, the frequency of an ASA score of 1 in terms of physical status presentation was significantly higher in the long-term survivor group (62.3% [38/61] vs. 30.6% [11/36], *p* = 0.006) (Table 1). Regarding the tumor characteristics, patients in the long-term survivor group had smaller tumors (*p* = 0.037) and lower FNCLCC grades (*p* = 0.021). The histological types differed between the 2 groups; myxofibrosarcoma was more frequent in the long-term survival group (*p* = 0.086), whereas leiomyosarcoma was more frequent in the short-term survival group (*p* = 0.036). Regarding the treatment characteristics, patients in the short-term survivor group had more histologically positive margins (*p* = 0.024) and fewer postoperative radiotherapies administered (*p* = 0.001) than the patients in the long-term survival group (Table 2).

**Table 1:** Patient, tumor and treatment characteristics of the study

	Long-term survivors (*n* = 61)	Short-term survivors (*n* = 36)	*P-value*
**Patient characteristics**			
Age (years)	48 (13–82)	59.5 (19–83)	n.s.
Sex (male)	32 (52.5%)	22 (61.1%)	n.s.
ASA score			**0.006**
I	38 (62.3%)	11 (30.6%)	
II	21 (34.4%)	23 (63.9%)	
III	2 (3.3%)	2 (5.6%)	
**Tumor characteristics**			
Location (extremity)	50 (82.0%)	28 (77.8%)	n.s.
Initial presentation			
Unplanned excision	5 (8.2%)	1 (2.8%)	n.s.
Size			
> 10 cm	22 (36.1%)	21 (58.3%)	**0.037**
FNCLCC Grade			
3	29 (47.5%)	26 (72.2%)	**0.021**
Depth			
Superficial	5 (8.2%)	1 (2.8%)	n.s.
Histological subtypes			
Leiomyosarcoma	3 (4.9%)	7 (19.4%)	**0.036**
**Treatment characteristics**			
Surgical			
Marginal	9 (14.8%)	9 (25%)	n.s.
Microscopic margin			
R1	3 (4.9%)	6 (16.7%)	**0.024**
Administration of adjuvant chemotherapy	18 (29.5%)	13 (36.1%)	n.s.
Administration of adjuvant radiotherapy	55 (90.2%)	21 (58.3%)	**0.001**

*n.s.* Not significant, *ASA* American Society of Anesthesiologists, *FNCLCC* Fédération Nationale des Centres de Lutte Contre le Cancer, *R1* Microscopically positive surgical margin

**Table 2:** Histological subtypes of long-term and short-term survivor groups

	Long-term survivors	Short-term survivors	*P-value*
**Pathology**			
UPS	19	9	0.644
Synovial sarcoma	10	6	1.000
Myxofibrosarcoma	9	1	0.086
Liposarcoma	7	2	0.477
Fibrosarcoma	4	1	0.648
MPNST	3	4	0.418
Leiomyosarcoma	3	7	**0.036**
Extraskeletal chondrosarcoma	3	1	1.000
Myxoid liposarcoma	1	0	1.000
Malignant hemangiopericytoma	1	0	1.000
Extraskeletal osteosarcoma	1	1	1.000
Epithelioid sarcoma	0	2	0.135
Extraskeletal Ewing’s sarcoma	0	1	0.375
Clear cell sarcoma	0	1	0.375

*UPS* Undifferentiated pleomorphic sarcoma, *MPNST* Malignant peripheral nerve sheath tumor

### Factors associated with actual long-term survival

On multivariate logistic regression analysis, factors independently associated with long-term survival were low ASA scores [odds ratio (OR) 11.3, p = 0.001], small tumor sizes (OR 3.9, *p* = 0.047), and low histological grades (OR 8.1, *p* = 0.004) (Table 3).

**Table 3:** Multivariate analysis of factors associated with actual long-term survival

	Odds Ratio	95% CI	*P-value*
ASA score 1	11.25	1.81–12.66	**0.001**
FNCLCC grade 2	8.12	1.45–10.64	**0.004**
Tumor size ≤10 cm	3.95	1.00–6.41	**0.047**

*CI* Confidence interval, *ASA* American Society of Anesthesiologists, *FNCLCC* Fédération Nationale des Centres de Lutte Contre le Cancer

Histological grade were independently associated with overall survival in multivariate actuarial analysis (Supplementary Table A, *p* = 0.015). On the contrary, tumor size and lower ASA score were not significantly related to survival in the actuarial analysis.

### Comparison of recurrence between long-term and short-term survivors

When the recurrence rates in the two groups were compared, only about 10% of the long-term survivors developed recurrence, while all the short-term survivors developed recurrence (Table 4). The recurrence timings were also quite different between the two groups. In contrast with 67% of those who developed recurrence within 1 year in the short-term survivor group, only one of the long-term survivors (14%) developed recurrence within 1 year (*p* = 0.009). Metastatic recurrence was also more common in the short-term survivor group (69.4% vs. 28.6%). Moreover, patients in the short-term survivor group underwent less aggressive treatment against recurrence compared to long-term survivors among which only 9 patients (25%) underwent surgical resection of recurred tumors. Conversely, all the long-term survivors who developed recurrence underwent subsequent surgical resection of the recurred tumors.

**Table 4:** Tumor recurrence details of the study patients

	Long-term survivors (*n* = 7)	Short-term survivors (*n* = 36)	*P-value*
**Recurrence timing**			**0.009**
< 1 year	1 (14.3%)	24 (66.7%)	
1-2 years	2 (28.6%)	7 (19.4%)	
2-5 years	3 (42.9%)	5 (13.9%)	
> 5 years	1 (14.3%)	0 (0.0%)	
**Recurrence location**			**0.04**
Regional	5 (71.4%)	11 (30.6%)	
Metastatic	2 (28.6%)	25 (69.4%)	
**Recurrence treatment**			**< 0.001**
Resection	3 (42.9%)	5 (13.9%)	
Resection + chemo/radiation	4 (57.1%)	4 (11.1%)	
Chemo/radiation only	0 (0.0%)	10 (27.8%)	
Supportive only	0 (0.0%)	17 (47.2%)	

*n.s.* Not significant

## Discussion

In this study, we compared 61 patients with AJCC stage III STS who survived for more than 5 years with 36 patients who died of the disease within 5 years. Lower ASA scores, smaller tumor sizes, and lower histological tumor grades were each independently associated with better outcomes. However, these risk factors did not preclude long-term survival, as 22 of 43 patients (51.2%) with tumor sizes larger than 10 cm and 29 of 55 (52.7%) patients with grade 3 tumors were long-term survivors.

The tumor size is subdivided and emphasized in the new 8th AJCC STS staging system. Grade 2 or 3 sarcomas larger than 5 cm are classified as stage III and those larger than 10 cm are subclassified as stage IIIB. Likewise, in the present study, tumors larger than 10 cm were associated with poor long-term survival. Contrary to the new AJCC staging system in which grade 2 or 3 does not alter the tumor stage, grade 3 was significantly associated with poor survival in this study.

The ASA score, ranging from 1 to 6, was developed to predict the operative risk of patients with certain physiological statuses [16]. ASA 1 stands for a group of healthy patients without any systemic disease including hypertension and diabetes mellitus, among others. Many previous studies [20, 21] have suggested a relationship between the ASA score and oncological outcomes, including those of STS. Likewise, in this study, low ASA scores were significantly associated with long-term survival. This finding suggests that the individual’s general physiological status can independently affect the oncological outcome. Analysis with a more commonly used measure of performance status, such as the Eastern Cooperative Oncology Group (ECOG) performance status, might have been useful. The Eastern Cooperative Oncology Group (ECOG) performance status were available in only about half of the study patients.

The actual survival rate in our study was 62.9% (61 of 97). The estimated survival rate of patients with AJCC stage III STS was 50% in a previous study [14]. However, this previous study was based on the 7th AJCC staging system. In a relatively new study [22] using the 8th AJCC staging system, the disease-specific survival rates for stage IIIA and IIIB STS were 77 and 62%, respectively, which were higher than the findings of this study (72 and 51%, respectively). The authors expected the actual survival rate to be somewhat lower than the estimated survival rate since the Kaplan–Meier method tends to overestimate the survival probability for cancers with poor prognosis [15]. However, in our study, these were very similar (62.9% vs. 62.8%). In retrospect, the prognosis of patients with stage III STS may not be as poor as that implied by the exaggerated estimated survival rate, unlike those corresponding to pancreatic cancer, hepatocellular carcinoma, and adrenocortical carcinoma [15, 23, 24]. Unlike histological grade, tumor size and ASA grade were independently associated with longer survival only in the actual survival analysis (not in actuarial analysis). This discrepancy may be validated in a future study on a larger population.

Leiomyosarcoma and malignant peripheral nerve sheath tumor (MPNST) were poor prognostic histology according to a previous study regarding prognostic factors of STS [7]. We observed a similar tendency in 7 of 10 patients with leiomyosarcoma and 4 of 7 patients with MPNST who were short-term survivors. In contrast, 9 of 10 patients with myxofibrosarcoma survived for more than 5 years in our study. Previous studies [25, 26] have shown better prognosis of myxofibrosarcoma in comparison to other types of sarcomas, although with higher local recurrence rates.

The administration of adjuvant radiotherapy was associated with long-term survival in the univariate analysis. Fifteen of the 36 short-term survivors did not undergo adjuvant radiotherapy; 4 presented with early distant metastasis, 3 had a poor general condition, and one of them had prolonged wound-healing problem. The remaining 7 patients did not receive adjuvant radiotherapy, as decided by radiation oncologists considering the tumor histology. The survival advantage of adjuvant radiotherapy, in part, reflects the difference in tumor biology apart from the benefit of radiotherapy itself [27].

Unplanned excision of STSs leads to poor outcomes compared to those of planned excision [28, 29]. It is without doubt that unplanned excision causes high morbidity and may lead to poor functional outcomes; however, its relation with disease-specific survival remains unclear. A previous study [30] has shown non-inferior oncological outcomes in patients who underwent aggressive re-excisions after unplanned excision of stage III STS. No conclusion could be made in our study because only a small number of patients with stage III STS (*n* = 6) were referred to our institute after unplanned excision. However, five of six patients who underwent unplanned excisions survived for more than 5 years in our study. The effect of unplanned excision on oncological outcomes in patients with AJCC stage III STS remains to be validated.

While all of the short-term survivors developed tumor recurrence in our study, only about 10% of the long-term survivors developed recurrence. Comparing the recurrence timing between the two groups, early recurrence within a year after resection was more common in the short-term survival group (67% vs. 14%). This finding may imply the importance of tumor surveillance, especially during the first year after resection, since early recurrence may lead to poor survival [31]. However, instead of early recurrence, micrometastasis may already have been present at the time of initial surgery, thus leading to early recurrence and poor survival outcomes [32]. Developing this idea, patients exhibiting factors of “poor” prognosis may benefit more from adjuvant chemotherapy, the only effective treatment against micrometastasis, because these patients may already have micrometastasis before resection. The higher rate of metastatic recurrence in the short-term survivor group in our study supports this hypothesis. Selection of these patients with “poor” prognosis for adjuvant chemotherapy remains to be studied in the future.

Surgical resection after tumor recurrence may improve the survival rate in patients with stage III STS according to our study. Although many other factors including individual comorbidities, age, tumor location, and the presence of distant metastasis must be considered in deciding whether to perform surgical resection of recurred tumors, our finding suggests that long-term survival is possible with adequate treatment including surgical resection even in cases of recurrence. More studies with larger patient groups must be conducted in the future to verify this finding.

The results of our study must be carefully interpreted considering some limitations. First, we performed a retrospective study of patients treated at a single tertiary referral hospital. Future validations are needed using external databases in a prospective setting. Second, the drop-out rate might have been higher in the short-term survivor group because the deceased patients may have discontinued visiting the hospital without notice. For the purpose of our study, the authors excluded the censored data from the analysis, and this may have caused an overestimation of the actual survival rate.

## Conclusion

Actual 5-year survival after resection of stage III STS was adversely associated with high ASA scores, large tumor sizes, and high histological grades. However, more than 65% of 5-year survivors possessed these poor prognostic factors, suggesting that aggressive treatment should be offered for a chance of long-term survival in these patients.

## Supplementary Information

**Additional file 1 Supplementary Table A.** Univariate and multivariate analysis on the actuarial survival of stage III soft tissue sarcoma.

## Data Availability

The dataset(s) supporting the conclusions of this article is(are) included within the article (and its additional file(s)).

## Abbreviations

STS: Soft tissue sarcoma
OR: Odds ratio
ASA: American Society of Anesthesiologicsts
AJCC: American Joint Committee on Cancer
FNCLCC: Fédération Nationale des Centres de Lutte Contre Le Cancer
MRI: Magnetic resonance imaging
US: Ultrasonography
ECOG: Eastern Cooperative Oncology Group
MPNST: Malignant peripheral nerve sheath tumor
