# Supplementary material for: Actual long-term survival after resection of stage III soft tissue sarcoma
Source: BMC Cancer. 2021 Jan 5;21:21. doi: 10.1186/s12885-020-07730-3 (PMC7786893; doi:10.1186/s12885-020-07730-3)
Supplement: Supplementary file 1 — Additional file 1 Supplementary Table A. Univariate and multivariate analysis on the actuarial survival of stage III soft tissue sarcoma. [file 12885_2020_7730_MOESM1_ESM.docx]

**Supplementary Table A. Univariate and multivariate analysis on the actuarial survival of stage III soft tissue sarcoma**

|  | Univariate | | | | | | | Multivariate | | | | | |  |  |
| --- | --- | --- | --- | --- | --- | --- | --- | --- | --- | --- | --- | --- | --- | --- | --- |
|  | 5y overall survival | | P | | 5y relapse-free survival | | P | OR (overall) | | P | OR (relapse-free) | | P |  |  |
| Age (years)  < 50  ≥50 | 17-49 **^a^**  51-81 **^a^** | 0.100 | | 17-49 **^a^**  51-67 **^a^** | | 0.241 | |  |  | |  |  | |  |  |
| Sex  Female  Male | 59.8 ± 7.2  61.6 ± 6.3 | 0.806 | | 56.8 ± 7.4  50.0 ± 7.1 | | 0.838 | |  |  | |  |  | |  |  |
| ASA score  I  II  III | 72.7 ± 6.0  49.4 ± 7.4  50.0 ± 25.0 | 0.095 | | 68.0 ± 6.4  38.3 ± 7.8  37.5 ± 28.6 | | **0.044** | |  |  | |  |  | |  |  |
| Initial presentation  Unplanned excision  Planned excision | 55.6 ± 16.6  61.3 ± 4.9 | 0.809 | | 44.4 ± 16.6  54.1 ± 5.3 | | 0.694 | |  |  | |  |  | |  |  |
| Size  ≤ 10cm  >10cm | 66.7 ± 5.8  52.8 ± 7.8 | 0.288 | | 58.0 ± 6.4  46.8 ± 8.2 | | 0.245 | |  |  | |  |  | |  |  |
| FNCLCC Grade  2  3 | 71.9 ± 6.7  51.0 ± 6.4 | **0.015** | | 57.5 ± 7.9  49.4 ± 6.6 | | 0.131 | | 1  8.31 | **0.015** | |  |  | |  |  |
| Microscopic margin  R0  R1 | 62.2 ± 4.9  57.1 ± 18.7 | 0.161 | | 55.7 ± 5.2  22.2 ± 19.2 | | 0.331 | |  |  | |  |  | |  |  |
| Adjuvant chemotherapy  (+)  (-) | 75.9 ± 7.9  55.9 ± 5.6 | 0.080 | | 73.9 ± 8.6  46.4 ± 6.0 | | **0.020** | |  |  | |  |  | |  |  |
| Administration of adjuvant radiotherapy  (+)  (-) | 70.6 ± 5.6  46.7 ± 7.4 | **0.009** | | 64.4 ± 6.3  35.0 ± 8.1 | | **0.006** | |  |  | |  |  | |  |  |

OR, odds ratio; **^a^** , ages are presented as ranges to insure patient anonymity; ASA, American Society of Anesthesiologists; FNCLCC, Fédération Nationale des Centres de Lutte Contre le Cancer; R0, microscopically negative surgical margin; R1, microscopically positive surgical margin
